# Development of a machine-learning algorithm to predict in-hospital cardiac arrest for emergency department patients using a nationwide database

**DOI:** 10.1038/s41598-022-26167-1

**Published:** 2022-12-16

**Authors:** Ji Hoon Kim, Arom Choi, Min Joung Kim, Heejung Hyun, Sunhee Kim, Hyuk-Jae Chang

**Affiliations:** 1 Department of Emergency Medicine, Yonsei University College of Medicine, 50-1 Yonsei-ro, Seodaemun-gu, Seoul, 03722 Republic of Korea; 2 AITRICS, 28 Hyoryeongro77-gil, Seocho-gu, Seoul, 06627 Republic of Korea; 3 CONNECT-AI Research Center, Yonsei University College of Medicine, 50-1 Yonsei-ro, Seodaemun-gu, Seoul, 03722 Republic of Korea; 4 Department of Cardiology, Yonsei University College of Medicine, 50-1 Yonsei-ro, Seodaemun-gu, Seoul, 03722 Republic of Korea

**Keywords:** Predictive medicine, Computational models

## Abstract

In this retrospective observational study, we aimed to develop a machine-learning model using data obtained at the prehospital stage to predict in-hospital cardiac arrest in the emergency department (ED) of patients transferred via emergency medical services. The dataset was constructed by attaching the prehospital information from the National Fire Agency and hospital factors to data from the National Emergency Department Information System. Machine-learning models were developed using patient variables, with and without hospital factors. We validated model performance and used the SHapley Additive exPlanation model interpretation. In-hospital cardiac arrest occurred in 5431 of the 1,350,693 patients (0.4%). The extreme gradient boosting model showed the best performance with area under receiver operating curve of 0.9267 when incorporating the hospital factor. Oxygen supply, age, oxygen saturation, systolic blood pressure, the number of ED beds, ED occupancy, and pulse rate were the most influential variables, in that order. ED occupancy and in-hospital cardiac arrest occurrence were positively correlated, and the impact of ED occupancy appeared greater in small hospitals. The machine-learning predictive model using the integrated information acquired in the prehospital stage effectively predicted in-hospital cardiac arrest in the ED and can contribute to the efficient operation of emergency medical systems.

## Introduction

Critically ill patients usually arrive at the emergency department (ED) through emergency medical services (EMS). Paramedics must quickly recognize the patient’s condition, and transport them to the optimal hospital, while providing appropriate first aid. Several studies have been conducted to develop a prehospital prediction tool for differentiating general patients from patients who are critically ill or have specific conditions, such as myocardial infarction or stroke, that require immediate treatment^1–4^. Since the ED and EMS are increasingly overloaded, and ambulance diversion is frequent, it is important for paramedics to accurately select and transport critically ill patients to the appropriate hospital to save their lives and support the efficient operation of medical facilities^5–7^.

With the growing demand for high-quality healthcare, embedding artificial intelligence (AI) into healthcare systems is a solution which promises to improve productivity and efficiency^8,9^. Since in-hospital cardiac arrest (IHCA) has a low survival rate, and is a major public healthcare burden that causes intensive consumption of medical resources, it is valuable to predict and minimize the occurrence of IHCA^10,11^. Recently, several studies that used AI to predict IHCA through patient clinical features have reported that AI-based models are superior to conventional rule-based tools^12–14^. However, to our knowledge, no studies have been conducted to predict the occurrence of IHCA at the prehospital stage. Because it can be challenging to assess information and make accurate and objective decisions in chaotic prehospital scenes, automated AI predictive models can be used to help paramedics make optimal decisions. In addition, the quality of care in the ED is adversely affected by crowding and overwhelmed medical staff. Previous studies have also reported that ED crowding increases the incidence of IHCA^10,15^. Therefore, the patient’s clinical features and hospital conditions should be considered to predict IHCA occurrence. AI can be an effective tool to play an auxiliary role in decision-making by integrating such a wide range of information.

In this study, we developed a machine-learning (ML) model to predict the occurrence of IHCA in the ED of patients arriving via EMS. Our research hypothesis was that an ML model that includes both patient clinical data and hospital factors could effectively predict the occurrence of IHCA at the prehospital stage.

## Methods

### Study design and setting

We conducted a retrospective, observational cohort study using a nationwide dataset that matched the National Fire Agency (NFA) data to the National Emergency Department Information System (NEDIS) in Korea. The Korean NFA is responsible for responding to fire, disaster, rescue, and EMS, following the Framework Act on Fire Services. The NFA has service headquarters in 18 cities and provinces and operates 210 fire stations. When paramedics transfer an emergency patient from the scene to the ED, they fill out a transfer record using a nationwide unified form and submit it to the NFA. The transfer record is written and transmitted in electronic form through a device owned by paramedics and managed by the NFA. Since 2013, the transfer record by paramedics has been amended four times by the Expert Quality Management Committee. This committee conducts audit and quality control of the NFA data registry and operates a data-based prehospital quality management program.

Hospitals must transmit emergency patient information via the NEDIS, a computerized system that collects data, such as clinical information and treatment results, of patients visiting ED nationwide. This system is managed by the National Emergency Medical Center (NEMC). Hospitals are also obligated to periodically transmit data related to ED crowding, such as the number of beds and occupants. This crowding information is disclosed online in real time for public purposes. Our study data were constructed by attaching the NFA’s prehospital information and ED crowding status at the time of patient arrival to the data of each patient registered in the NEDIS.

This study included patients transported to the ED by EMS and had information transmitted to the NEDIS from September 2017 to December 2018. Children younger than 18 years of age, and those who experienced cardiac arrest before ED arrival, were excluded from the study. Data were provided anonymously by the NFA and NEMC, and the work was performed in a secure manner on a computer that only the researchers in this study had access to. The institutional review boards of Severance Hospital permitted us to proceed with the study, including an exemption from obtaining the patient’s informed consent (4-2021-0580). This research was conducted in accordance with the principles in the Declaration of Helsinki.

### Data processing

The NEMC recommends that crowding data be automatically transmitted at least once every 15 min or at least once every hour for manual transmissions. We obtained NEDIS data for our sample, including the patients’ age and sex, arrival time and treatment codes at the ED. The number of hospitals, beds, and patients occupying the ED at arrival was added to the matched patient data. We selected the crowding data transmitted closest to the patient’s arrival at the ED.

The NFA data included the following variables: patient’s age, sex, category of non-medical problems (e.g. hanging, trauma, or poisoning), medical history, symptoms, level of consciousness, vital signs (blood pressure, pulse rate, body temperature, and oxygen saturation), blood sugar level, emergency care provided during transport (cardiopulmonary resuscitation (CPR), laryngeal mask airway, oxygen administration, and intravenous infusion of fluid), phone call time, ED arrival time, area of occurrence, and the name of the hospital to which the patient was transferred. Hypo- and hyperglycemia were defined as blood sugar levels below 80 mg/dL and above 250 mg/dL, respectively.

We matched the NFA data to the NEDIS data based on patient age, sex and arrival time. We accepted a 10-min difference for the arrival time of the two datasets. If two or more patients in the NFA data were matched for one patient in the NEDIS database, the records were matched to the hospital name.

Since the NEDIS data contained information on the treatment performed in the ED, we used it to determine the occurrence of sudden IHCA requiring resuscitation. The electronic data interchange code M1583-7, corresponding to CPR, was used to define the IHCA group. Patients were excluded if the NFA data indicated CPR before arrival at the hospital. ED occupancy rate was used as an indicator of crowding. We calculated the occupancy rate by dividing the number of occupants by the number of beds^16^. Although there are no globally agreed representative indicator for measuring ED crowding, the occupancy rate is one of the most promising methods for quantification^17^.

### Model development

The complete dataset was randomly split into training and test sets in an 8:2 ratio. We used the training set to develop the prediction model for IHCA. Two datasets were generated: one containing all variables, including hospital factors, and a second model containing only patient factors (excluding hospital factors). We trained three ML models: logistic regression (LR), extreme gradient boosting (XGB, XGBoost), and multilayer perceptron network (MLP). The training set was split into 10 folds. We performed grid-search for hyperparameter tuning and the details of search space and selected setting are in Supplementary Table 1. In order to resolve the data imbalance problem, we applied naïve random sampling in training folds where the validation fold and test set were untouched. First, we oversampled IHCA positive samples to raise the class ratio by 10%. Next, we under-sampled negative samples to change the class ratio by 30%. After random sampling, the size of training folds was shrunk to 30% of the original total training folds. The area under the receiver operating characteristic curve (AUROC) and area under the precision-recall curve (AUPRC) were calculated in the test fold in every epoch. We used the highest AUROC score among every ten iterations to select the single best model from LR, XGB, and MLP.

### Model validation

The performances of the LR, XGB and MLP models with and without hospital factors were validated in the test set. Model performance was estimated using AUROC, AUPRC, sensitivity, specificity, accuracy, positive predictive value (PPV), and negative predictive value (NPV) with 95% confidence intervals. We compared the AUROC values of different model algorithms, and selected the final model with the highest AUROC value.

In order to interpret the final predictive model, we adopted the SHapley Additive exPlanation (SHAP) proposed by Lundberg and Lee in 2017^18^. SHAP can explain any ML model’s output by calculating the impact of each feature on model prediction based on game theory. SHAP allows us to understand which feature is the most important to model prediction, and the positive or negative direction of the feature impact. Applying SHAP to our developed model, we used the DeepExplainer module, which enables the fast approximation of SHAP values in the deep learning model, and the TreeExplainer module, which is an optimized SHAP algorithm for tree ensemble methods such as XGB^19^. First, we ranked the feature importance by SHAP values. We calculated the AUROC score in the test set, starting from feature values all set by zero-value, sequentially adding one feature to replace feature values from zero to their own data. We confirmed the increasing trend of AUROC when variables were added one by one in the order of the most influential variables.

Considering that the patient characteristics and frequency of IHCA could differ depending on the size of the hospital, additional validation was carried out in four subgroups defined by the quartile of the number of hospital beds. We confirmed the SHAP value of ED crowding according to hospital size by visualizing the dependence plot to evaluate the impact of the hospital factors.

### Statistical analysis

Continuous variables were presented as the mean and standard deviation and compared between groups using Student’s *t* test. Nominal variables are expressed in frequency and fraction and analyzed using the Chi-square test. We developed models using three ML methods, LR, XGB and MLP, and compared the AUROC of each model using the DeLong test to determine whether the difference between models was statistically significant^20^. Meanwhile, the significance of the AUPRC difference was calculated by a bootstrap of 1000 iterations. Estimations of confidence intervals by single model prediction were obtained from Hanley JA for AUROC, Boyd for AUPRC, Wilson score interval for sensitivity, specificity, and accuracy, and Mercaldo ND for PPV and NPV^21–24^. The optimal cut-off value was calculated using Jouden’s index. We calibrated the final model by platt scaling and isotonic regression and checked the Brier score. All statistical analyses were implemented and performed in Python with the SciPy and scikit-learn packages, and p-values < 0.05 were considered statistically significant.

## Results

**Figure 1:**
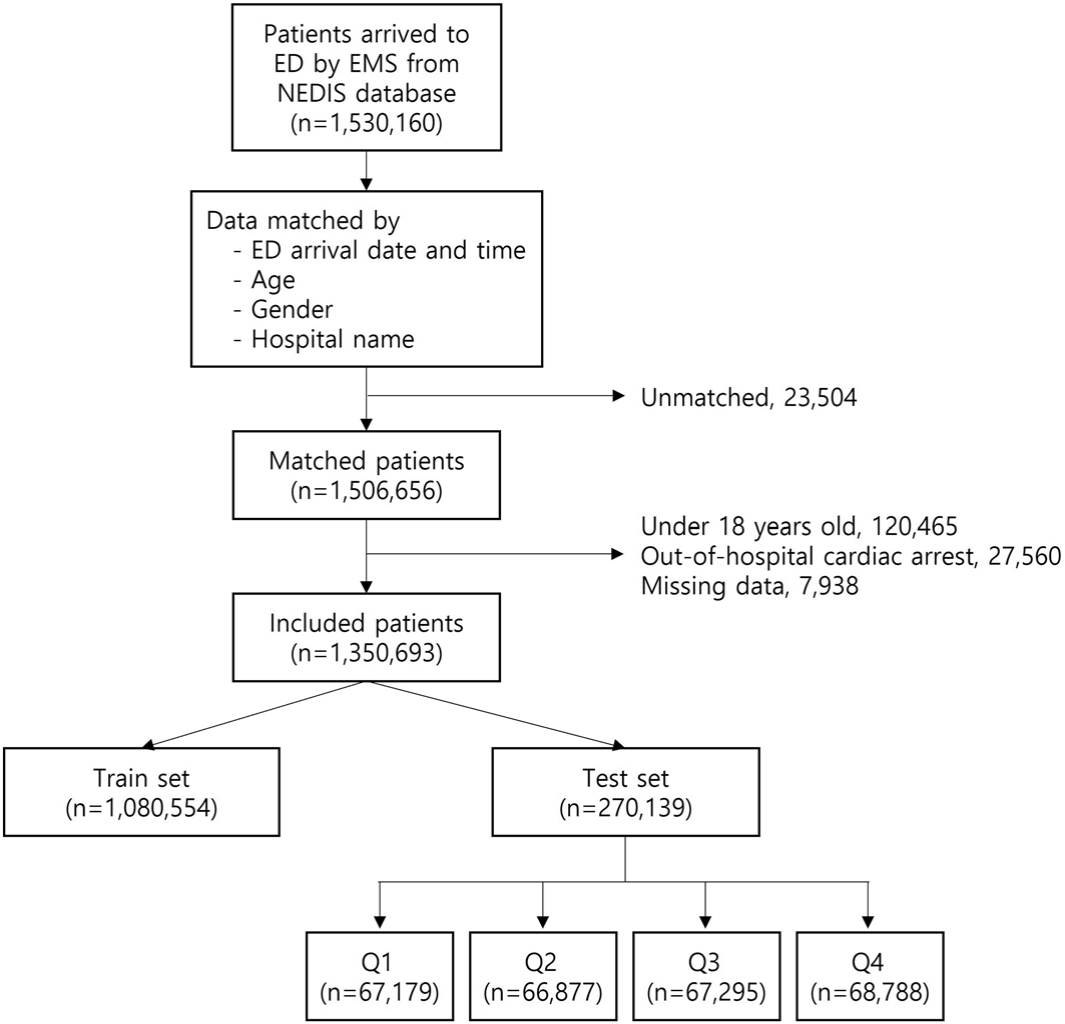
Included patients. *ED* emergency department, *EMS* emergency medical service, *NEDIS* National Emergency Department Information System.

We identified 1,530,160 patients from the NEDIS who arrived nationwide at the ED via EMS during the study period (Fig. 1). Within that group, 23,504 patients did not match the NFA data. Among 1,506,656 patients who succeeded in matching, we excluded 120,465 patients under 18, 27,560 patients with out-of-hospital cardiac arrest, and 7938 patients with missing data. Our final data set consisted of 1,350,693 eligible patients. The training set had 1,080,554 individuals, and the test set consisted of 270,139 individuals. The clinical characteristics of the patients in the training and test sets were similar and are presented in Supplementary Table 2. IHCA occurred in 5431 patients (0.4%). Patients in the IHCA group were significantly older (70.13 ± 15.50 vs. 57.98 ± 19.09, p < 0.001) and had a lower proportion of men than those in the non-IHCA group (Table 1). The IHCA group had a higher mean number of hospital beds (761.65 ± 462.17 vs. 621.97 ± 441.80, p < 0.001) and higher ED occupancy (0.63 ± 0.51 vs. 0.45 ± 0.36, p < 0.001).

**Table 1:** Comparison of patient characteristics between IHCA and non-IHCA group.

Variables		Total (n = 1,350,693)	IHCA (n = 5431)	Non-IHCA (n = 1,345,262)	p-value
Male gender		636,175 (47.10)	2088 (38.45)	634,087 (47.13)	< 0.001
Age		58.03 ± 19.09	70.13 ± 15.50	57.98 ± 19.09	< 0.001
Non-medical problem		465,531 (34.47)	742 (13.66)	464,789 (34.55)	< 0.001
Medical history	Hypertension	368,997 (27.32)	1683 (30.99)	367,314.0 (27.30)	< 0.001
	Diabetes mellitus	213,650 (15.82)	1165 (21.45)	212,485.0 (15.80)	< 0.001
	Heart disease	100,119 (7.41)	711 (13.09)	99,408.0 (7.39)	< 0.001
	Brain disease	71,871 (5.32)	484 (8.91)	71,387.0 (5.31)	< 0.001
	Cancer	70,343 (5.21)	913 (16.81)	69,430.0 (5.16)	< 0.001
	Lung disease	29,485 (2.18)	324 (5.97)	29,161.0 (2.17)	< 0.001
	Renal failure	20,633 (1.53)	203 (3.74)	20,430.0 (1.52)	< 0.001
	Liver cirrhosis	16,125 (1.19)	241 (4.44)	15,884.0 (1.18)	< 0.001
	Hepatitis	3075 (0.23)	18 (0.33)	3057.0 (0.23)	0.108
	Tuberculosis	2138 (0.16)	18 (0.33)	2120.0 (0.16)	0.001
	Allergy	1910 (0.14)	4 (0.07)	1906.0 (0.14)	0.183
	Other disease	303,485 (22.47)	2038 (37.53)	301,447.0 (22.41)	< 0.001
Symptom	Other pain	395,188 (29.26)	592 (10.90)	394,596 (29.33)	< 0.001
	Nausea and vomiting	174,088 (12.89)	453 (8.34)	173,635 (12.91)	< 0.001
	Abdominal pain	169,553 (12.55)	436 (8.03)	169,117 (12.57)	< 0.001
	General weakness	163,428 (12.10)	1053 (19.39)	162,375 (12.07)	< 0.001
	Laceration	106,514 (7.89)	226 (4.16)	106,288 (7.90)	< 0.001
	Dizziness	93,868 (6.95)	119 (2.19)	93,749 (6.97)	< 0.001
	Flank pain	91,052 (6.74)	111 (2.04)	90,941 (6.76)	< 0.001
	Headache	81,742 (6.05)	75 (1.38)	81,667 (6.07)	< 0.001
	Abrasion	69,210 (5.12)	151 (2.78)	69,059 (5.13)	< 0.001
	Fever	62,188 (4.60)	254 (4.68)	61,934 (4.60)	0.798
	Bleeding	55,438 (4.10)	294 (5.41)	55,144 (4.10)	< 0.001
	Dyspnea	55,096 (4.08)	1399 (25.76)	53,697 (3.99)	< 0.001
	Contusion	48,928 (3.62)	66 (1.22)	48,862 (3.63)	< 0.001
	Mental change	48,203 (3.57)	1681 (30.95)	46,522 (3.46)	< 0.001
	Chest pain	42,247 (3.13)	416 (7.66)	41,831 (3.11)	< 0.001
	diarrhea	38,918 (2.88)	108 (1.99)	38,810 (2.88)	< 0.001
	Syncope	17,843 (1.32)	77 (1.42)	17,766 (1.32)	0.531
	Fracture	17,172 (1.27)	116 (2.14)	17,056 (1.27)	< 0.001
	Side weakness	16,634 (1.23)	37 (0.68)	16,597 (1.23)	< 0.001
	Epistaxis	16,010 (1.19)	71 (1.31)	15,939 (1.18)	0.405
	Seizure	13,768 (1.02)	53 (0.98)	13,715 (1.02)	0.749
	Voiding difficulty	10,647 (0.79)	21 (0.39)	10,626 (0.79)	< 0.001
	Sprain	10,620 (0.79)	5 (0.09)	10,615 (0.79)	< 0.001
	Cough	9546 (0.71)	26 (0.48)	9520 (0.71)	0.044
	Hematemesis	8927 (0.66)	261 (4.81)	8666 (0.64)	< 0.001
	Psychosis	6629 (0.49)	7 (0.13)	6622 (0.49)	< 0.001
	Tachycardia	6408 (0.47)	15 (0.28)	6393 (0.48)	0.033
	Dislocation	5256 (0.39)	5 (0.09)	5251 (0.39)	< 0.001
	Constipation	4731 (0.35)	7 (0.13)	4724 (0.35)	0.006
	Convulsion	4149 (0.31)	17 (0.31)	4132 (0.31)	0.938
	Burn	3143 (0.23)	3 (0.06)	3140 (0.23)	0.007
	Hypothermia	2359 (0.17)	43 (0.79)	2316 (0.17)	< 0.001
	Hemoptysis	2310 (0.17)	28 (0.52)	2282 (0.17)	< 0.001
	Extremities weakness	2043 (0.15)	8 (0.15)	2035 (0.15)	0.940
	Vaginal bleeding	1970 (0.15)	2 (0.04)	1968 (0.15)	0.035
	Other foreign body	1489 (0.11)	4 (0.07)	1485 (0.11)	0.416
	Airway foreign body	1297 (0.10)	27 (0.50)	1270 (0.09)	< 0.001
	Compartment	921 (0.07)	5 (0.09)	916 (0.07)	0.499
	Amputation	853 (0.06)	2 (0.04)	851 (0.06)	0.439
	Labor pain	395 (0.03)	2 (0.04)	393 (0.03)	0.743
	Others	246,151 (18.22)	916 (16.87)	245,235 (18.23)	0.009
Mental status	Alert	1,256,230 (93.01)	2905 (53.49)	1,253,325 (93.17)	< 0.001
	Verbal	50,435 (3.73)	739 (13.61)	49,696 (3.69)	< 0.001
	Pain	36,699 (2.72)	1091 (20.09)	35,608 (2.65)	< 0.001
	Unresponsive	7306 (0.54)	694 (12.78)	6612 (0.49)	< 0.001
Vital sign	Systolic blood pressure	132.01 ± 24.50	112.34 ± 34.91	132.08 ± 24.42	< 0.001
	Diastolic blood pressure	82.39 ± 16.12	71.35 ± 23.39	82.43 ± 16.07	< 0.001
	Pulse rate	85.62 ± 17.76	94.10 ± 28.74	85.59 ± 17.69	< 0.001
	Respiratory rate	17.85 ± 4.28	19.69 ± 6.31	17.84 ± 4.27	< 0.001
	Body temperature	37.82 ± 8.37	37.69 ± 8.61	37.82 ± 8.37	0.252
	Oxygen saturation	97.02 ± 5.33	86.48 ± 13.64	97.06 ± 5.23	< 0.001
Blood sugar	Hypoglycemia	3751 (0.28)	37 (0.68)	3714 (0.28)	< 0.001
	Hyperglycemia	4188 (0.31)	54 (0.99)	4134 (0.31)	< 0.001
Emergency care	Laryngeal mask airway	675 (0.05)	80 (1.47)	595 (0.04)	< 0.001
	Oxygen administration	183,903 (13.62)	3913 (72.05)	179,990 (13.38)	< 0.001
	Intravenous fluid infusion	29,345 (2.17)	711 (13.09)	28,634 (2.13)	< 0.001
Day of arrival	Monday	199,311 (14.76)	866 (15.95)	198,445 (14.75)	0.013
	Tuesday	187,454 (13.88)	756 (13.92)	186,698 (13.88)	0.929
	Wednesday	187,152 (13.86)	725 (13.35)	186,427 (13.86)	0.279
	Thursday	187,108 (13.85)	718 (13.22)	186,390 (13.86)	0.177
	Friday	192,685 (14.27)	767 (14.12)	191,918 (14.27)	0.763
	Saturday	199,923 (14.80)	807 (14.86)	199,116 (14.80)	0.905
	Sunday	197,060 (14.59)	792 (14.58)	196,268 (14.59)	0.989
Hour of arrival	0	54,370 (4.03)	172 (3.17)	54,198 (4.03)	0.001
	1	47,824 (3.54)	161 (2.96)	47,663 (3.54)	0.021
	2	41,447 (3.07)	146 (2.69)	41,301 (3.07)	0.104
	3	36,808 (2.73)	117 (2.15)	36,691 (2.73)	0.010
	4	33,296 (2.47)	136 (2.50)	33,160 (2.46)	0.853
	5	33,841 (2.51)	161 (2.96)	33,680 (2.50)	0.030
	6	38,036 (2.82)	202 (3.72)	37,834 (2.81)	< 0.001
	7	47,209 (3.50)	244 (4.49)	46,965 (3.49)	< 0.001
	8	60,012 (4.44)	291 (5.36)	59,721 (4.44)	0.001
	9	72,559 (5.37)	321 (5.91)	72,238 (5.37)	0.078
	10	72,488 (5.37)	322 (5.93)	72,166 (5.36)	0.066
	11	66,660 (4.94)	286 (5.27)	66,374 (4.93)	0.259
	12	62,318 (4.61)	276 (5.08)	62,042 (4.61)	0.099
	13	61,435 (4.55)	238 (4.38)	61,197 (4.55)	0.556
	14	61,921 (4.58)	261 (4.81)	61,660 (4.58)	0.435
	15	61,195 (4.53)	225 (4.14)	60,970 (4.53)	0.169
	16	60,570 (4.48)	234 (4.31)	60,336 (4.49)	0.531
	17	59,906 (4.44)	268 (4.93)	59,638 (4.43)	0.073
	18	63,341 (4.69)	253 (4.66)	63,088 (4.69)	0.914
	19	67,091 (4.97)	268 (4.93)	66,823 (4.97)	0.912
	20	63,486 (4.70)	239 (4.40)	63,247 (4.70)	0.296
	21	62,923 (4.66)	232 (4.27)	62,691 (4.66)	0.175
	22	62,635 (4.64)	202 (3.72)	62,433 (4.64)	0.001
	23	59,322 (4.39)	176 (3.24)	59,146 (4.40)	< 0.001
Time from call to ED arrival		27.19 ± 12.87	28.69 ± 13.24	27.18 ± 12.87	< 0.001
Area	Gyeonggi	314,987 (23.32)	1329 (24.47)	313,658 (23.32)	0.045
	Seoul	293,835 (21.75)	1064 (19.59)	292,771 (21.76)	< 0.001
	Inchoen	91,070 (6.74)	281 (5.17)	90,789 (6.75)	< 0.001
	Busan	80,320 (5.95)	407 (7.49)	79,913 (5.94)	< 0.001
	Gyeongbuk	71,509 (5.29)	226 (4.16)	71,283 (5.30)	< 0.001
	Chungnam	59,358 (4.39)	170 (3.13)	59,188 (4.40)	< 0.001
	Jeonbuk	53,817 (3.98)	265 (4.88)	53,552 (3.98)	< 0.001
	Daegu	52,475 (3.89)	470 (8.65)	52,005 (3.87)	< 0.001
	Gangwon	52,084 (3.86)	246 (4.53)	51,838 (3.85)	0.010
	Jeonnam	49,521 (3.67)	129 (2.38)	49,392 (3.67)	< 0.001
	Daejeon	49,496 (3.66)	152 (2.80)	49,344 (3.67)	< 0.001
	Gyeongnam	47,450 (3.51)	242 (4.46)	47,208 (3.51)	< 0.001
	Chungbuk	46,594 (3.45)	159 (2.93)	46,435 (3.45)	0.035
	Gwangju	34,105 (2.53)	149 (2.74)	33,956 (2.52)	0.304
	Jeju	32,995 (2.44)	86 (1.58)	32,909 (2.45)	< 0.001
	Ulsan	21,077 (1.56)	56 (1.03)	21,021 (1.56)	0.002
Hospital factor	Hospital bed	622.53 ± 441.97	761.65 ± 462.17	621.97 ± 441.80	< 0.001
	ED bed	26.73 ± 14.94	32.34 ± 15.25	26.71 ± 14.94	< 0.001
	ED occupancy	0.45 ± 0.36	0.63 ± 0.51	0.45 ± 0.36	< 0.001

*IHCA* in-hospital cardiac arrest, *ED* emergency department.

### Model validation

**Figure 2:**
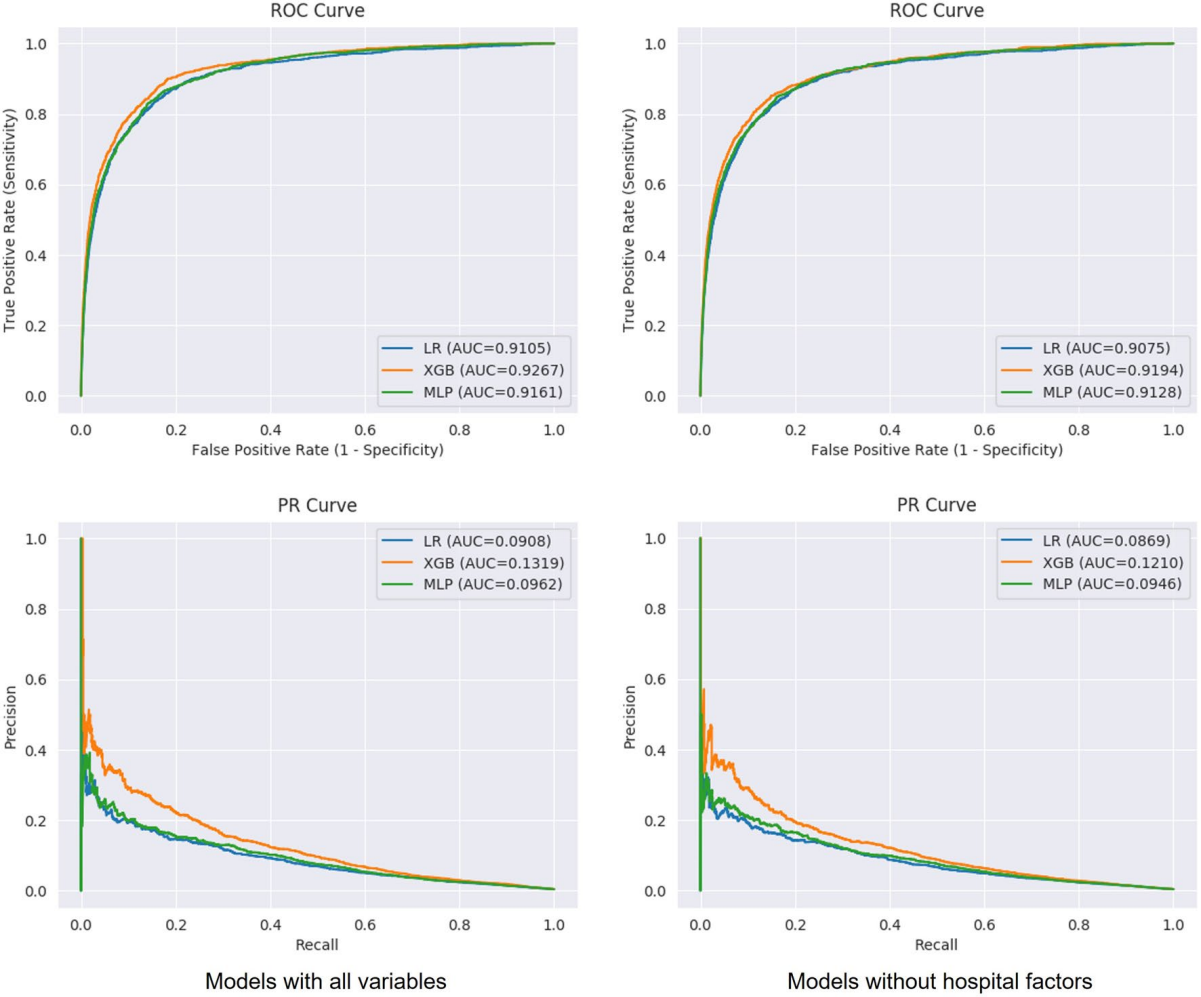
Performance of machine-learning models to predict the occurrence of in-hospital cardiac arrest. *ROC* receiver operating characteristic, *PR* precision-recall, *AUC* area under the curve, *LR* logistic regression, *XGB* extreme gradient boosting, *MLP* multilayer perceptron network.

The performances of the models were evaluated using a test set, and ML models with a combination of naïve random over/under-sampling did not show better performance (Supplementary Table 3). Figure 2 shows the ROC and PR curves of models predicting the occurrence of IHCA developed using LR, XGB, and MLP. The AUROC of all three ML models showed high performance with 0.9 or higher, and XGB, including the hospital factor, was the highest at 0.9267. In AUPRC, the value of the XGB model, including all variables, was 0.1319, the highest compared with LR and MLP. The comparative analysis of AUROC and AUPRC between the models are shown in Supplementary Table 4. We used logistic regression and isotonic regression for probability calibration and the calibrators were fit with validation fold. We selected the ML model of the XGB method, including all variables, as the final model. The Brier score of the final XGB model was 0.0037 and the calibration plot is shown in Supplementary Fig. 1.

The performance of the final model was verified using four subgroups of the test set. The test set was divided into quartiles (Q1–Q4) bordering 296, 568, and 818 of the number of hospital beds. From Q1 to Q4, there were more patients with dyspnea, chest pain, and mental change, and more emergency care was performed at the site. The incidence of IHCA in the ED was also higher (Supplementary Table 5). The AUROC of the model’s prediction of IHCA occurrence was evaluated to be 0.9 or higher in all subgroups, and the AUPRC increased from Q1 to Q4 and was the highest at 0.1532 in Q4 (Supplementary Fig. 2). Supplementary Table 6 presents the performance analysis results of the subgroups.

### Model interpretation

**Figure 3:**
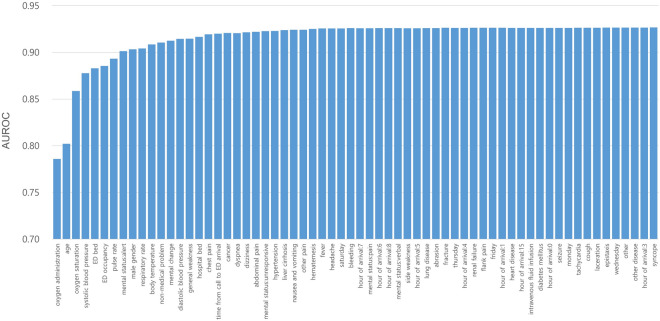
Parsimony plot of variables and predictive performance of model using extreme gradient boosting. *AUROC* area under the receiver operating characteristic curve, *ED* emergency department.

The parsimony plot in Fig. 3 shows the trend of increasing the AUROC of the final model when variables were added one by one in the top-ranking order. The largest increase in model performance was observed from the first to the eighth variables, after which marginal gains in performance were added as the remaining variables were input. The most influential variable was oxygen supply, followed by age, oxygen saturation, and systolic blood pressure. The number of ED beds and ED occupancy were the fifth and sixth most influential variables in the model performance, respectively.

**Figure 4:**
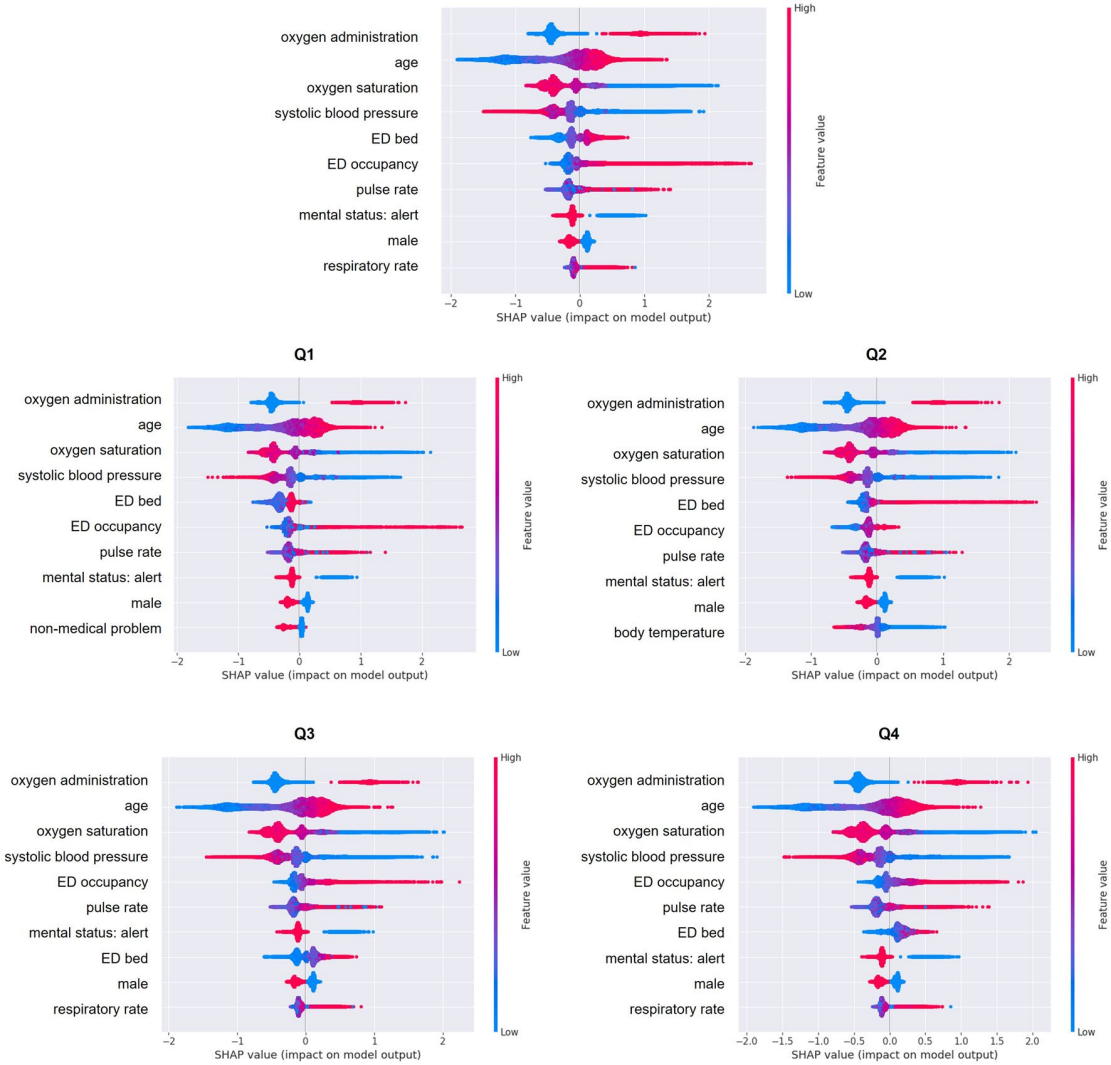
SHapley Additive exPlanation of model predicting the occurrence of in-hospital cardiac arrest in the test set. *SHAP* SHapley Additive exPlanation, *ED* emergency department.

**Figure 5:**
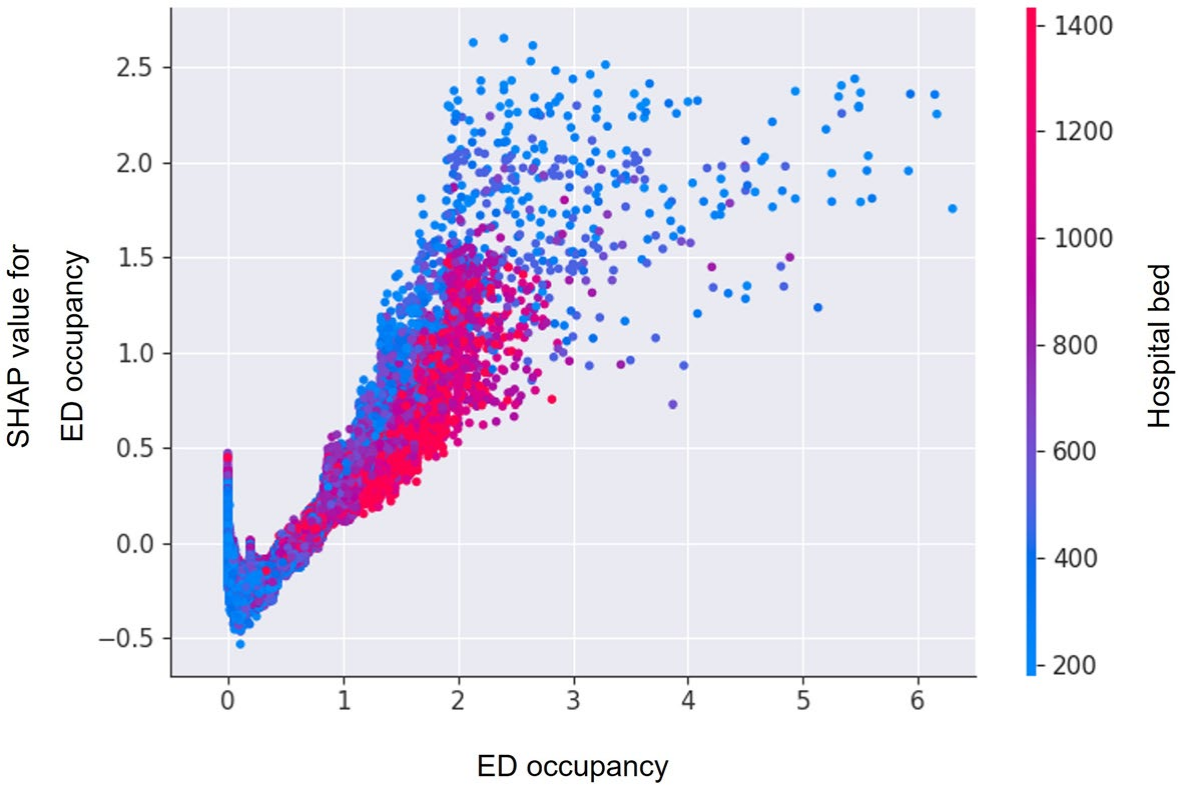
Dependence plot of the degree of influence of ED occupancy on the occurrence of in-hospital cardiac arrest according to the number of hospital beds. *SHAP* SHapley Additive explanation, *ED* emergency department.

Figure 4 shows the SHAP of the variables when the model was applied to the test set. The top ten variables with the most influence on the model are shown on the y-axis in rank order. As shown in the parsimony plot, oxygen supply, age, oxygen saturation, and systolic blood pressure were the top four most influential variables in the test set, which was also confirmed in the subgroups. ED occupancy was selected as the 5th or 6th most influential variable, and ED occupancy and IHCA occurrence were positively correlated in large and small hospitals. Figure 5 shows a dependence plot of the SHAP value of ED occupancy according to the number of hospital beds. Compared with hospitals with many beds, the SHAP value of ED occupancy tends to be higher in small hospitals.

## Discussion

In this study, we found that the ML-based predictive model using integrated information acquired in the prehospital stage effectively predicted the occurrence of IHCA in the ED. We also confirmed that hospital factors such as ED crowding, were the main factors in the prediction model. The application of ML for decision-making is gradually expanding in the medical field, including during the prehospital stage^25,26^. However, while the data generated during the prehospital stage are not yet fully utilized in ML research, it is significant that we developed a predictive model with integrated information based on the national dataset extracted from the standardized prehospital care system.

All three machine-learning algorithms trained in our study showed AUROC values above 0.9 for ED IHCA prediction. The disadvantage of the imbalanced dataset is that the AUROC is optimistic; therefore, all our models yielded high AUROC results, which were statistically significant but with minimal numerical differences. Therefore, it is desirable to consider AUPRC along with AUROC for model performance. AUPRC was 0.13 in XGB, which was higher than that of LR and MLP. The AUPRC is a single number summary of the information in the precision-recall curve, and is a useful performance metric for imbalanced data in a setting focused on finding positive examples^24,27^. While the baseline of AUROC is always 0.5, AUPRC is a relative indicator because the baseline is the proportion of positive cases to the population^24^. In our dataset, which has 0.004 as the baseline AUPRC, an AUPRC value of 0.132 by the XGB algorithm reflects favorable performance in IHCA prediction. XGB, a tree ensemble model, has been reported to perform better in classification and regression problems involving tabular data organized in rows and columns, which are the most common data types in traditional statistical modeling^28–30^.

The disadvantage of our model was that the AUPRC and PPV were very low due to the imbalanced dataset. Therefore, when applied to the real world, the problem of false alarms is inevitable. However, we expect that our predictive model has high sensitivity and can be used for the purpose of screening patients with the potential to develop IHCA. IHCA in the ED is sudden and unpredictable, but considered to be preventable if early identification of at-risk patients and adequate interventions are possible^15,31^. Therefore, a predictive model for screening patients at the emergency scene and transporting them to the optimal hospital will help reduce the incidence of IHCA and increase the survival of patients.

Machine learning-based predictive tools may raise doubts about their clinical applicability because they do not provide explanations supported by clinical relevance^9,13^. In this study, to overcome the black-box aspect of machine learning, we tried to present clinical validity by using an explanatory machine learning called SHAP. Based on the SHAP analysis, we found that clinical variables associated with the occurrence of IHCA (i.e. oxygen supply and saturation, vital signs, and mental status) in previous studies were in line with variables that had a high influence on the output of the present model^32–34^. In addition to these key factors, our model included more clinical data such as medical history, symptoms, first aid, and hospital factors, increasing the predictive power over traditional statistical methods. Making the optimal choice from such a wide range of information is an advantage of AI. However, these various data must be input in real time to apply AI models in an emergency scene, which cannot be done using a conventional manual system. Automated collection and rapid processing of real-time integrated data must be available to ensure the feasibility of predictive models.

Along with variables reflecting the patient’s medical condition, hospital factors such as the number of ED beds and ED occupancy also ranked highly as significant predictors of IHCA. These results were found consistently across all subgroups divided based on hospital size. A comparison of the patient characteristics of the subgroups showed that large hospitals have numerous ED beds, more crowded ED, and a higher frequency of IHCA. In the dependence plot analyzed to consider the relationship between these variables, we found a positive correlation between ED crowding and its impact on IHCA incidence in hospitals of all sizes. ED crowding is associated with a lack of resources for patients needing immediate resuscitation^35^. Previous studies have reported that ED crowding is associated with the occurrence of IHCA^10,15^. In the dependence plot, we could also confirm that ED crowding had a greater effect on the occurrence of IHCA in small hospitals than in large hospitals. In the case of ED crowding, small hospitals do not have sufficient medical resources available and are less able to cope with crises than large hospitals. For this reason, paramedics usually decide to transfer seriously ill patients in urgent situations to a larger hospital, but the level of crowding is often not considered. Although the ED crowding status is shared online, it is not easy for paramedics to manually search for this information in emergencies. As ED crowding is a factor that affects the quality of care, it is necessary to establish a process that can be considered for the selection of an appropriate transfer hospital by quickly obtaining crowding information when transporting emergency patients. In addition, because ED crowding causes ambulance diversion, paramedics can use this process to reduce the retransfer of emergent patients^36^.

Our study had several limitations. First, our study included the potential bias of its retrospective design. Second, as this study was conducted in a single country, caution is needed when generalizing the study results. Finally, the present predictive model has not been validated in the real world. In particular, since our predictive model has suboptimal performance metrics due to imbalanced data, the usefulness of the developed model needs to be verified through additional prospective studies before being applied to prehospital care. Establishing a digital platform to deploy the ML algorithm developed in this study is necessary to demonstrate its usefulness prospectively.

## Conclusions

The ML-based predictive model developed by integrating various data, including hospital factors and patients’ clinical information generated in the prehospital stage, effectively predicted the occurrence of IHCA. The AI decision support system will enable evidence-based judgment with increased utilization of the information available in emergencies, thereby providing the basis for the efficient use of emergency medical resources while ensuring patient safety.

## Supplementary Information

Supplementary Information.

## Data Availability

The data that support the findings of this study are available from the National Fire Agency (NFA) and the National Emergency Medical Center (NEMC) but restrictions apply to the availability of these data, which were used under license for the current study, and so are not publicly available. Data are however available from the authors upon reasonable request and with permission of NFA and NEMC.

## Abbreviations

AI: Artificial intelligence
AUROC: Area under the receiver operating characteristic curve
AUPRC: Area under the precision-recall curve
CPR: Cardiopulmonary resuscitation
ED: Emergency department
EMS: Emergency medical services
IHCA: In-hospital cardiac arrest
LR: Logistic regression
ML: Machine learning
MLP: Multilayer perceptron network
NEDIS: National Emergency Department Information System
NEMC: National Emergency Medical Center
NFA: National Fire Agency
NPV: Negative predictive value
PPV: Positive predictive value
SHAP: SHapley Additive exPlanation
XGB: Extreme gradient boosting
