# Supplementary material for: Development of a machine-learning algorithm to predict in-hospital cardiac arrest for emergency department patients using a nationwide database
Source: Sci Rep. 2022 Dec 16;12:21797. doi: 10.1038/s41598-022-26167-1 (PMC9758227; doi:10.1038/s41598-022-26167-1)
Supplement: Supplementary file 1 — Supplementary Information. [file 41598_2022_26167_MOESM1_ESM.docx]

**Supplementary information**

**Development of a machine-learning algorithm to predict in-hospital cardiac arrest for emergency department patients using a nationwide database**

Ji Hoon Kim, MD, MPH, PhD^a†^; Arom Choi, MD^a†^; Min Joung Kim, MD, PhD^a*^; Heejung Hyun, MS^b^; Sunhee Kim, MS^c^; Hyuk-Jae Chang MD, PhD^d^

^a^Department of Emergency Medicine, Yonsei University College of Medicine, 50 Yonsei-ro, Seodaemun-gu, Seoul 03722, Republic of Korea

^b^AITRICS, 28 Hyoryeongro77-gil, Seocho-gu, Seoul 06627, Republic of Korea

^c^CONNECT-AI research center, Severance Hospital, Yonsei University College of Medicine, Seodaemun-gu, 50-1 Yonsei-ro, Seoul 03722, Republic of Korea

^d^Department of Cardiology, Yonsei University College of Medicine, 50 Yonsei-ro, Seodaemun-gu, Seoul 03722, Republic of Korea

†Ji Hoon Kim and Arom Choi contributed equally to this work.

**^*^Corresponding author:**

Min Joung Kim, MD, PhD

Department of Emergency Medicine, Yonsei University College of Medicine, 50 Yonsei-ro, Seodaemun-gu, Seoul 03722, Republic of Korea

**Supplementary Table 1. Details of model hyperparameter tuning**

| Model hyperparameter search space for grid-search | |
| --- | --- |
| XGB | eta: [0.1, 0.05, 0.01, 0.001] |
|  | max_depth: [2, 3, 4, 5, 8] |
|  | num_rounds: [200, 500, 1000, 10000] |
|  | tree_method: [‘auto’, ‘gpu_hist’] |
| MLP | initial learning rate: [1e-2, 1e-3, 1e-4, 1e-5] |
|  | batch size: [128, 256, 512, 1024, 2048] |
|  | num_epochs: [300, 400, 500] |
|  | optimizer: [‘SGD’, ‘Adam’] |
|  | weight_decay: [1e-4, 1e-5] |
|  | weighted cross entropy loss: [True, False] |
| Final tested models’ hyper-parameters | |
| XGB | Both models with and without hospital factors have same hyperparameters |
|  | eta: 0.05 / max_depth: 4 / num_rounds: 500 / tree_method: ‘gpu_hist’ |
| MLP | Both models with and without hospital factors have 3-hidden layers composed by following sequence of structures. |
|  | Layer1: Dense(64) + Batch Normalization + ELU (activation) |
|  | Layer2: Dense(128) + Batch Normalization + ELU (activation) + Dropout(0.25) |
|  | Layer3: Dense(256) + Batch Normalization + ELU (activation) + Dropout(0.5) |
|  | Hyperparameters |
|  | Initial lr: 1e-2 / batch: 2048 / num_epochs: 300 / optimizer: SGD / weight decay: 1e-4 / no weighted cross entropy loss |

XGB, extreme gradient boosting; MLP, multilayer perceptron network; SGD, stochastic gradient descent; ELU, exponential linear unit

**Supplementary Table 2. Comparison of patient characteristics between training and test set**

| Variables |  | Total (n=1350693) | Training (n=1080554) | Test (n=270139) | p-value |
| --- | --- | --- | --- | --- | --- |
| Male gender |  | 636175 (47.10) | 508866 (47.09) | 127309 (47.13) | 0.750 |
| Age |  | 58.03±19.09 | 58.04±19.09 | 58.01±19.09 | 0.424 |
| Non-medical problem | | 465531 (34.47) | 372265 (34.45) | 93266 (34.53) | 0.470 |
| Medical history | hypertension | 368997 (27.32) | 295416 (27.34) | 73581 (27.24) | 0.292 |
|  | diabetes mellitus | 213650 (15.82) | 171073 (15.83) | 42577 (15.76) | 0.367 |
|  | heart disease | 100119 (7.41) | 80076 (7.41) | 20043 (7.42) | 0.875 |
|  | brain disease | 71871 (5.32) | 57462 (5.32) | 14409 (5.33) | 0.739 |
|  | cancer | 70343 (5.21) | 56309 (5.21) | 14034 (5.20) | 0.738 |
|  | lung disease | 29485 (2.18) | 23615 (2.19) | 5870 (2.17) | 0.691 |
|  | renal failure | 20633 (1.53) | 16579 (1.53) | 4054 (1.50) | 0.203 |
|  | liver cirrhosis | 16125 (1.19) | 12843 (1.19) | 3282 (1.21) | 0.259 |
|  | hepatitis | 3075 (0.23) | 2485 (0.23) | 590 (0.22) | 0.259 |
|  | tuberculosis | 2138 (0.16) | 1738 (0.16) | 400 (0.15) | 0.135 |
|  | allergy | 1910 (0.14) | 1524 (0.14) | 386 (0.14) | 0.819 |
|  | other disease | 303485 (22.47) | 242795 (22.47) | 60690 (22.47) | 0.971 |
| Symptom | other pain | 395188 (29.26) | 315940 (29.24) | 79248 (29.34) | 0.320 |
|  | nausea and vomiting | 174088 (12.89) | 139158 (12.88) | 34930 (12.93) | 0.471 |
|  | abdominal pain | 169553 (12.55) | 135514 (12.54) | 34039 (12.60) | 0.405 |
|  | general weakness | 163428 (12.10) | 130622 (12.09) | 32806 (12.14) | 0.427 |
|  | laceration | 106514 (7.89) | 85059 (7.87) | 21455 (7.94) | 0.225 |
|  | dizziness | 93868 (6.95) | 75063 (6.95) | 18805 (6.96) | 0.791 |
|  | flank pain | 91052 (6.74) | 72934 (6.75) | 18118 (6.71) | 0.428 |
|  | headache | 81742 (6.05) | 65515 (6.06) | 16227 (6.01) | 0.273 |
|  | abrasion | 69210 (5.12) | 55238 (5.11) | 13972 (5.17) | 0.205 |
|  | fever | 62188 (4.60) | 49879 (4.62) | 12309 (4.56) | 0.187 |
|  | bleeding | 55438 (4.10) | 44321 (4.10) | 11117 (4.12) | 0.750 |
|  | dyspnea | 55096 (4.08) | 44225 (4.09) | 10871 (4.02) | 0.107 |
|  | contusion | 48928 (3.62) | 39103 (3.62) | 9825 (3.64) | 0.650 |
|  | mental change | 48203 (3.57) | 38638 (3.58) | 9565 (3.54) | 0.381 |
|  | chest pain | 42247 (3.13) | 33767 (3.12) | 8480 (3.14) | 0.705 |
|  | diarrhea | 38918 (2.88) | 31253 (2.89) | 7665 (2.84) | 0.127 |
|  | syncope | 17843 (1.32) | 14215 (1.32) | 3628 (1.34) | 0.263 |
|  | fracture | 17172 (1.27) | 13797 (1.28) | 3375 (1.25) | 0.254 |
|  | side weakness | 16634 (1.23) | 13218 (1.22) | 3416 (1.26) | 0.082 |
|  | epistaxis | 16010 (1.19) | 12897 (1.19) | 3113 (1.15) | 0.077 |
|  | seizure | 13768 (1.02) | 10961 (1.01) | 2807 (1.04) | 0.253 |
|  | voiding difficulty | 10647 (0.79) | 8528 (0.79) | 2119 (0.78) | 0.800 |
|  | sprain | 10620 (0.79) | 8518 (0.79) | 2102 (0.78) | 0.592 |
|  | cough | 9546 (0.71) | 7648 (0.71) | 1898 (0.70) | 0.774 |
|  | hematemesis | 8927 (0.66) | 7147 (0.66) | 1780 (0.66) | 0.886 |
|  | psychosis | 6629 (0.49) | 5278 (0.49) | 1351 (0.50) | 0.438 |
|  | tachycardia | 6408 (0.47) | 5125 (0.47) | 1283 (0.47) | 0.965 |
|  | dislocation | 5256 (0.39) | 4181 (0.39) | 1075 (0.40) | 0.411 |
|  | constipation | 4731 (0.35) | 3795 (0.35) | 936 (0.35) | 0.710 |
|  | convulsion | 4149 (0.31) | 3291 (0.30) | 858 (0.32) | 0.273 |
|  | burn | 3143 (0.23) | 2502 (0.23) | 641 (0.24) | 0.580 |
|  | hypothermia | 2359 (0.17) | 1858 (0.17) | 501 (0.19) | 0.133 |
|  | hemoptysis | 2310 (0.17) | 1855 (0.17) | 455 (0.17) | 0.716 |
|  | extremities weakness | 2043 (0.15) | 1650 (0.15) | 393 (0.15) | 0.388 |
|  | vaginal bleeding | 1970 (0.15) | 1561 (0.14) | 409 (0.15) | 0.398 |
|  | other foreign body | 1489 (0.11) | 1192 (0.11) | 297 (0.11) | 0.959 |
|  | airway foreign body | 1297 (0.10) | 1046 (0.10) | 251 (0.09) | 0.560 |
|  | compartment | 921 (0.07) | 721 (0.07) | 200 (0.07) | 0.193 |
|  | amputation | 853 (0.06) | 685 (0.06) | 168 (0.06) | 0.824 |
|  | labor pain | 395 (0.03) | 313 (0.03) | 82 (0.03) | 0.706 |
|  | others | 246151 (18.22) | 197009 (18.23) | 49142 (18.19) | 0.623 |
| Mental status | alert | 1256230 (93.01) | 1004961 (93.00) | 251269 (93.01) | 0.849 |
|  | verbal | 50435 (3.73) | 40245 (3.72) | 10190 (3.77) | 0.243 |
|  | pain | 36699 (2.72) | 29513 (2.73) | 7186 (2.66) | 0.042 |
|  | unresponsive | 7306 (0.54) | 5816 (0.54) | 1490 (0.55) | 0.398 |
| Vital sign | systolic blood pressure | 132.01±24.50 | 132.01±24.49 | 131.98±24.54 | 0.550 |
|  | diastolic blood pressure | 82.39±16.12 | 82.40±16.12 | 82.36±16.14 | 0.328 |
|  | pulse rate | 85.62±17.76 | 85.61±17.76 | 85.66±17.76 | 0.247 |
|  | respiratory rate | 17.85±4.28 | 17.85±4.29 | 17.85±4.27 | 0.883 |
|  | body temperature | 37.82±8.37 | 37.82±8.39 | 37.80±8.31 | 0.151 |
|  | oxygen saturation | 97.02±5.33 | 97.02±5.33 | 97.02±5.33 | 0.498 |
| Blood sugar | hypoglycemia | 3751 (0.28) | 3024 (0.28) | 727 (0.27) | 0.343 |
|  | hyperglycemia | 4188 (0.31) | 3403 (0.31) | 785 (0.29) | 0.042 |
| Emergency care | laryngeal mask airway | 675 (0.05) | 511 (0.05) | 164 (0.06) | 0.005 |
|  | oxygen administration | 183903 (13.62) | 147238 (13.63) | 36665 (13.57) | 0.468 |
|  | intravenous fluid infusion | 29345 (2.17) | 23603 (2.18) | 5742 (2.13) | 0.061 |
| Day of arrival | Monday | 199311 (14.76) | 159578 (14.77) | 39733 (14.71) | 0.433 |
|  | Tuesday | 187454 (13.88) | 150020 (13.88) | 37434 (13.86) | 0.724 |
|  | Wednesday | 187152 (13.86) | 149737 (13.86) | 37415 (13.85) | 0.923 |
|  | Thursday | 187108 (13.85) | 149718 (13.86) | 37390 (13.84) | 0.844 |
|  | Friday | 192685 (14.27) | 154145 (14.27) | 38540 (14.27) | 0.986 |
|  | Saturday | 199923 (14.80) | 159721 (14.78) | 40202 (14.88) | 0.188 |
|  | Sunday | 197060 (14.59) | 157635 (14.59) | 39425 (14.59) | 0.937 |
| Hour of arrival | 0 | 54370 (4.03) | 43540 (4.03) | 10830 (4.01) | 0.630 |
|  | 1 | 47824 (3.54) | 38148 (3.53) | 9676 (3.58) | 0.196 |
|  | 2 | 41447 (3.07) | 33335 (3.08) | 8112 (3.00) | 0.027 |
|  | 3 | 36808 (2.73) | 29295 (2.71) | 7513 (2.78) | 0.046 |
|  | 4 | 33296 (2.47) | 26568 (2.46) | 6728 (2.49) | 0.340 |
|  | 5 | 33841 (2.51) | 27082 (2.51) | 6759 (2.50) | 0.899 |
|  | 6 | 38036 (2.82) | 30450 (2.82) | 7586 (2.81) | 0.783 |
|  | 7 | 47209 (3.50) | 37733 (3.49) | 9476 (3.51) | 0.689 |
|  | 8 | 60012 (4.44) | 48188 (4.46) | 11824 (4.38) | 0.063 |
|  | 9 | 72559 (5.37) | 58038 (5.37) | 14521 (5.38) | 0.930 |
|  | 10 | 72488 (5.37) | 57882 (5.36) | 14606 (5.41) | 0.301 |
|  | 11 | 66660 (4.94) | 53519 (4.95) | 13141 (4.86) | 0.058 |
|  | 12 | 62318 (4.61) | 50037 (4.63) | 12281 (4.55) | 0.061 |
|  | 13 | 61435 (4.55) | 49208 (4.55) | 12227 (4.53) | 0.536 |
|  | 14 | 61921 (4.58) | 49422 (4.57) | 12499 (4.63) | 0.238 |
|  | 15 | 61195 (4.53) | 48846 (4.52) | 12349 (4.57) | 0.255 |
|  | 16 | 60570 (4.48) | 48561 (4.49) | 12009 (4.45) | 0.275 |
|  | 17 | 59906 (4.44) | 47870 (4.43) | 12036 (4.46) | 0.567 |
|  | 18 | 63341 (4.69) | 50666 (4.69) | 12675 (4.69) | 0.945 |
|  | 19 | 67091 (4.97) | 53674 (4.97) | 13417 (4.97) | 0.990 |
|  | 20 | 63486 (4.70) | 50712 (4.69) | 12774 (4.73) | 0.435 |
|  | 21 | 62923 (4.66) | 50216 (4.65) | 12707 (4.70) | 0.212 |
|  | 22 | 62635 (4.64) | 50187 (4.64) | 12448 (4.61) | 0.419 |
|  | 23 | 59322 (4.39) | 47377 (4.38) | 11945 (4.42) | 0.398 |
| Time from call to ED arrival | | 27.19±12.87 | 27.19±12.87 | 27.17±12.87 | 0.572 |
| Area | Gyeonggi | 314987 (23.32) | 251857 (23.31) | 63130 (23.37) | 0.500 |
|  | Seoul | 293835 (21.75) | 235149 (21.76) | 58686 (21.72) | 0.673 |
|  | Inchoen | 91070 (6.74) | 72892 (6.75) | 18178 (6.73) | 0.757 |
|  | Busan | 80320 (5.95) | 64091 (5.93) | 16229 (6.01) | 0.134 |
|  | Gyeongbuk | 71509 (5.29) | 57211 (5.29) | 14298 (5.29) | 0.971 |
|  | Chungnam | 59358 (4.39) | 47579 (4.40) | 11779 (4.36) | 0.331 |
|  | Jeonbuk | 53817 (3.98) | 43120 (3.99) | 10697 (3.96) | 0.465 |
|  | Daegu | 52475 (3.89) | 41977 (3.88) | 10498 (3.89) | 0.974 |
|  | Gangwon | 52084 (3.86) | 41817 (3.87) | 10267 (3.80) | 0.094 |
|  | Jeonnam | 49521 (3.67) | 39721 (3.68) | 9800 (3.63) | 0.233 |
|  | Daejeon | 49496 (3.66) | 39561 (3.66) | 9935 (3.68) | 0.682 |
|  | Gyeongnam | 47450 (3.51) | 37806 (3.50) | 9644 (3.57) | 0.072 |
|  | Chungbuk | 46594 (3.45) | 37294 (3.45) | 9300 (3.44) | 0.825 |
|  | Gwangju | 34105 (2.53) | 27225 (2.52) | 6880 (2.55) | 0.419 |
|  | Jeju | 32995 (2.44) | 26421 (2.45) | 6574 (2.43) | 0.728 |
|  | Ulsan | 21077 (1.56) | 16833 (1.56) | 4244 (1.57) | 0.620 |
| Hospital factor | hospital bed | 622.53±441.97 | 622.61±442.14 | 622.20±441.30 | 0.667 |
|  | ED bed | 26.73±14.94 | 26.74±14.96 | 26.72±14.88 | 0.569 |
|  | ED occupancy | 0.45±0.36 | 0.45±0.36 | 0.45±0.36 | 0.737 |
| In-hospital cardiac arrest | | 5431 (0.40) | 4345 (0.40) | 1086 (0.40) | 0.995 |
| ED, emergency department | |  |  |  |  |

**Supplementary Table 3. Performance of models predicting the occurrence of in-hospital cardiac arrest with or without hospital factors**

| Model | Hospital factor | AUROC | AUPRC | Cutoff | Sensitivity | Specificity | Accuracy | PPV | NPV |
| --- | --- | --- | --- | --- | --- | --- | --- | --- | --- |
| LR | Y | 0.9105  (0.8988, 0.9223) | 0.0908  (0.0751, 0.1094) | 0.0034 | 0.8352  (0.8119, 0.8561) | 0.8446  (0.8432, 0.8460) | 0.8446  (0.8432, 0.8459) | 0.0212  (0.0207, 0.0218) | 0.9992  (0.9991, 0.9993) |
|  | N | 0.9075  (0.8956, 0.9194) | 0.0869  (0.0716, 0.1052) | 0.0026 | 0.8656  (0.8440, 0.8846) | 0.8082  (0.8068, 0.8097) | 0.8085  (0.8070, 0.8100) | 0.0179  (0.0172, 0.0186) | 0.9993  (0.9992, 0.9995) |
| XGB | Y | 0.9267  (0.9160, 0.9375) | 0.1319  (0.1131, 0.1534) | 0.0024 | 0.9006  (0.8813, 0.9170) | 0.8163  (0.8149, 0.8178) | 0.8167  (0.8152, 0.8181) | 0.0194  (0.0190, 0.0198) | 0.9995  (0.9994, 0.9996) |
|  | N | 0.9194  (0.9082, 0.9306) | 0.1210  (0.1029, 0.1418) | 0.0033 | 0.8517  (0.8294, 0.8716) | 0.8505  (0.8491, 0.8518) | 0.8505  (0.8491, 0.8518) | 0.0225  (0.0216, 0.0234) | 0.9993  (0.9991, 0.9995) |
| MLP | Y | 0.9161  (0.9047, 0.9275) | 0.0962  (0.0800, 0.1152) | 0.003 | 0.8610  (0.8391, 0.8803) | 0.8292  (0.8278, 0.8306) | 0.8293  (0.8279, 0.8308) | 0.0199  (0.0194, 0.0204) | 0.9993  (0.9992, 0.9994) |
|  | N | 0.9128  (0.9012, 0.9244) | 0.0946  (0.0785, 0.1135) | 0.0037 | 0.8490  (0.8265, 0.8691) | 0.8380  (0.8366, 0.8394) | 0.8381  (0.8367, 0.8395) | 0.0207  (0.0199, 0.0215) | 0.9993  (0.9991, 0.9994) |
| models with combination of naïve random over/under-sampling | | | | | | | | | |
| LR | Y | 0.9121  (0.9005, 0.9237) | 0.0819  (0.0670, 0.0998) | 0.2402 | 0.8674  (0.8459, 0.8863) | 0.8177  (0.8162, 0.8191) | 0.8179  (0.8164, 0.8193) | 0.0188  (0.0184, 0.0193) | 0.9993  (0.9992, 0.9994) |
|  | N | 0.9093  (0.8975, 0.9211) | 0.0815  (0.0666, 0.0993) | 0.2691 | 0.8407  (0.8177, 0.8613) | 0.8374  (0.8360, 0.8388) | 0.8374  (0.8360, 0.8388) | 0.0204  (0.0199, 0.0210) | 0.9992  (0.9991, 0.9993) |
| XGB | Y | 0.9257  (0.9148, 0.9365) | 0.1173  (0.0994, 0.1378) | 0.2703 | 0.8499  (0.8274, 0.8699) | 0.8622  (0.8609, 0.8635) | 0.8621  (0.8608, 0.8634) | 0.0243  (0.0237, 0.0249) | 0.9993  (0.9992, 0.9994) |
|  | N | 0.9187  (0.9074, 0.9299) | 0.1110  (0.0937, 0.1311) | 0.3024 | 0.8352  (0.8119, 0.8561) | 0.8644  (0.8631, 0.8657) | 0.8643  (0.8630, 0.8655) | 0.0243  (0.0236, 0.0249) | 0.9992  (0.9991, 0.9993) |
| MLP | Y | 0.9166  (0.9052, 0.9279) | 0.0865  (0.0712, 0.1048) | 0.2728 | 0.8536  (0.8313, 0.8734) | 0.8385  (0.8371, 0.8399) | 0.8386  (0.8372, 0.8400) | 0.0209  (0.0204, 0.0214) | 0.9993  (0.9992, 0.9994) |
|  | N | 0.9120  (0.9004, 0.9237) | 0.0857  (0.0704, 0.1039) | 0.3128 | 0.8481  (0.8255, 0.8682) | 0.8379  (0.8365, 0.8392) | 0.8379  (0.8365, 0.8393) | 0.0207  (0.0201, 0.0212) | 0.9993  (0.9992, 0.9994) |

AUROC, area under the receiver operating characteristic curve; AUPRC, area under the precision-recall curve; PPV, positive predictive value; NPV, negative predictive value; LR, logistic regression; XGB, extreme gradient boosting; MLP, multilayer perceptron network

**Supplementary Table 4. Comparison of AUROC and AUPRC between models predicting the occurrence of in-hospital cardiac arrest**

|  |  | with hospital factors | | | without hospital factors | | | with vs without hospital factors | | |
| --- | --- | --- | --- | --- | --- | --- | --- | --- | --- | --- |
| AUROC | comparison | LR vs XGB | LR vs MLP | XGB vs MLP | LR vs XGB | LR vs MLP | XGB vs MLP | LR | XGB | MLP |
|  | difference | 0.0162 | 0.0056 | 0.0106 | 0.0119 | 0.0054 | 0.0066 | 0.0031 | 0.0073 | 0.0033 |
|  | p-value | < 0.001 | 0.009 | < 0.001 | < 0.001 | 0.011 | 0.003 | 0.03 | < 0.001 | 0.100 |
| AUPRC | comparison | LR vs XGB | LR vs MLP | XGB vs MLP | LR vs XGB | LR vs MLP | XGB vs MLP | LR | XGB | MLP |
|  | difference | 0.0411 | 0.0054 | 0.0357 | 0.0341 | 0.0077 | 0.0264 | 0.0039 | 0.0109 | 0.0016 |
|  | p-value | 0.002 | 0.606 | 0.002 | 0.003 | 0.421 | 0.018 | 0.693 | 0.415 | 0.848 |

AUROC, area under the receiver operating characteristic curve; AUPRC, area under the precision-recall curve; LR, logistic regression; XGB, extreme gradient boosting; MLP, multilayer perceptron network

**Supplementary Table 5. Comparison of patient characteristics between subgroups based on the number of hospital beds**

| Variables |  | Test (n=270139) | Q1 (n=67179) | Q2 (n=66877) | Q3 (n=67295) | Q4 (n=68788) | p-value |
| --- | --- | --- | --- | --- | --- | --- | --- |
| Male gender |  | 127309 (47.13) | 31758 (47.27) | 31194 (46.64) | 31994 (47.54) | 32363 (47.05) | < 0.0001 |
| Age |  | 58.01±19.09 | 58.50±19.09 | 57.60±19.10 | 57.55±19.26 | 58.36±18.91 | < 0.0001 |
| Non-medical problem | | 93266 (34.53) | 26804 (39.90) | 23844 (35.65) | 22453 (33.37) | 20165 (29.31) | < 0.0001 |
| Medical history | hypertension | 73581 (27.24) | 17191 (25.59) | 17583 (26.29) | 18755 (27.87) | 20052 (29.15) | < 0.0001 |
|  | diabetes mellitus | 42577 (15.76) | 9961 (14.83) | 10352 (15.48) | 10742 (15.96) | 11522 (16.75) | < 0.0001 |
|  | heart disease | 20043 (7.42) | 3143 (4.68) | 4150 (6.21) | 5324 (7.91) | 7426 (10.80) | < 0.0001 |
|  | brain disease | 14409 (5.33) | 2609 (3.88) | 3271 (4.89) | 3779 (5.62) | 4750 (6.91) | < 0.0001 |
|  | cancer | 14034 (5.20) | 1878 (2.80) | 2419 (3.62) | 3540 (5.26) | 6197 (9.01) | < 0.0001 |
|  | lung disease | 5870 (2.17) | 1155 (1.72) | 1293 (1.93) | 1514 (2.25) | 1908 (2.77) | < 0.0001 |
|  | renal failure | 4054 (1.50) | 539 (0.80) | 787 (1.18) | 1196 (1.78) | 1532 (2.23) | < 0.0001 |
|  | liver cirrhosis | 3282 (1.21) | 547 (0.81) | 656 (0.98) | 991 (1.47) | 1088 (1.58) | < 0.0001 |
|  | hepatitis | 590 (0.22) | 86 (0.13) | 120 (0.18) | 168 (0.25) | 216 (0.31) | < 0.0001 |
|  | tuberculosis | 400 (0.15) | 56 (0.08) | 88 (0.13) | 116 (0.17) | 140 (0.20) | < 0.0001 |
|  | allergy | 386 (0.14) | 89 (0.13) | 84 (0.13) | 111 (0.16) | 102 (0.15) | 0.196 |
|  | other disease | 60690 (22.47) | 12010 (17.88) | 13170 (19.69) | 15983 (23.75) | 19527 (28.39) | < 0.0001 |
| Symptom | other pain | 79248 (29.34) | 22391 (33.33) | 20974 (31.36) | 18581 (27.61) | 17302 (25.15) | < 0.0001 |
|  | nausea and vomiting | 34930 (12.93) | 8190 (12.19) | 8689 (12.99) | 8770 (13.03) | 9281 (13.49) | < 0.0001 |
|  | abdominal pain | 34039 (12.60) | 8870 (13.20) | 8670 (12.96) | 8294 (12.32) | 8205 (11.93) | < 0.0001 |
|  | general weakness | 32806 (12.14) | 8130 (12.10) | 8215 (12.28) | 7653 (11.37) | 8808 (12.80) | < 0.0001 |
|  | laceration | 21455 (7.94) | 4561 (6.79) | 4587 (6.86) | 6168 (9.17) | 6139 (8.92) | < 0.0001 |
|  | dizziness | 18805 (6.96) | 3474 (5.17) | 4192 (6.27) | 5239 (7.79) | 5900 (8.58) | < 0.0001 |
|  | flank pain | 18118 (6.71) | 5650 (8.41) | 5053 (7.56) | 4042 (6.01) | 3373 (4.90) | < 0.0001 |
|  | headache | 16227 (6.01) | 3725 (5.54) | 3984 (5.96) | 4067 (6.04) | 4451 (6.47) | < 0.0001 |
|  | abrasion | 13972 (5.17) | 3947 (5.88) | 3457 (5.17) | 3357 (4.99) | 3211 (4.67) | < 0.0001 |
|  | fever | 12309 (4.56) | 2753 (4.10) | 2893 (4.33) | 3047 (4.53) | 3616 (5.26) | < 0.0001 |
|  | bleeding | 11117 (4.12) | 2244 (3.34) | 2330 (3.48) | 3123 (4.64) | 3420 (4.97) | < 0.0001 |
|  | dyspnea | 10871 (4.02) | 1676 (2.49) | 2182 (3.26) | 3155 (4.69) | 3858 (5.61) | < 0.0001 |
|  | contusion | 9825 (3.64) | 3032 (4.51) | 2470 (3.69) | 2248 (3.34) | 2075 (3.02) | < 0.0001 |
|  | mental change | 9565 (3.54) | 1492 (2.22) | 1968 (2.94) | 2846 (4.23) | 3259 (4.74) | < 0.0001 |
|  | chest pain | 8480 (3.14) | 1059 (1.58) | 1613 (2.41) | 2518 (3.74) | 3290 (4.78) | < 0.0001 |
|  | diarrhea | 7665 (2.84) | 2071 (3.08) | 2025 (3.03) | 1779 (2.64) | 1790 (2.60) | < 0.0001 |
|  | syncope | 3628 (1.34) | 596 (0.89) | 843 (1.26) | 1032 (1.53) | 1157 (1.68) | < 0.0001 |
|  | fracture | 3375 (1.25) | 930 (1.38) | 750 (1.12) | 885 (1.32) | 810 (1.18) | < 0.0001 |
|  | side weakness | 3416 (1.26) | 268 (0.40) | 593 (0.89) | 1116 (1.66) | 1439 (2.09) | < 0.0001 |
|  | epistaxis | 3113 (1.15) | 490 (0.73) | 544 (0.81) | 897 (1.33) | 1182 (1.72) | < 0.0001 |
|  | seizure | 2807 (1.04) | 365 (0.54) | 593 (0.89) | 840 (1.25) | 1009 (1.47) | < 0.0001 |
|  | voiding difficulty | 2119 (0.78) | 518 (0.77) | 503 (0.75) | 514 (0.76) | 584 (0.85) | 0.054 |
|  | sprain | 2102 (0.78) | 776 (1.16) | 589 (0.88) | 461 (0.69) | 276 (0.40) | < 0.0001 |
|  | cough | 1898 (0.70) | 455 (0.68) | 480 (0.72) | 448 (0.67) | 515 (0.75) | 0.122 |
|  | hematemesis | 1780 (0.66) | 168 (0.25) | 329 (0.49) | 568 (0.84) | 715 (1.04) | < 0.0001 |
|  | psychosis | 1351 (0.50) | 131 (0.20) | 237 (0.35) | 429 (0.64) | 554 (0.81) | < 0.0001 |
|  | tachycardia | 1283 (0.47) | 141 (0.21) | 201 (0.30) | 404 (0.60) | 537 (0.78) | < 0.0001 |
|  | dislocation | 1075 (0.40) | 335 (0.50) | 267 (0.40) | 275 (0.41) | 198 (0.29) | < 0.0001 |
|  | constipation | 936 (0.35) | 261 (0.39) | 257 (0.38) | 199 (0.30) | 219 (0.32) | 0.009 |
|  | convulsion | 858 (0.32) | 141 (0.21) | 167 (0.25) | 272 (0.40) | 278 (0.40) | < 0.0001 |
|  | burn | 641 (0.24) | 163 (0.24) | 175 (0.26) | 170 (0.25) | 133 (0.19) | 0.085 |
|  | hypothermia | 501 (0.19) | 153 (0.23) | 143 (0.21) | 103 (0.15) | 102 (0.15) | <0.001 |
|  | hemoptysis | 455 (0.17) | 45 (0.07) | 68 (0.10) | 112 (0.17) | 230 (0.33) | < 0.0001 |
|  | extremities weakness | 393 (0.15) | 45 (0.07) | 77 (0.12) | 117 (0.17) | 154 (0.22) | < 0.0001 |
|  | vaginal bleeding | 409 (0.15) | 22 (0.03) | 49 (0.07) | 140 (0.21) | 198 (0.29) | < 0.0001 |
|  | other foreign body | 297 (0.11) | 56 (0.08) | 50 (0.07) | 79 (0.12) | 112 (0.16) | < 0.0001 |
|  | airway foreign body | 251 (0.09) | 66 (0.10) | 54 (0.08) | 69 (0.10) | 62 (0.09) | 0.568 |
|  | compartment | 200 (0.07) | 45 (0.07) | 34 (0.05) | 70 (0.10) | 51 (0.07) | 0.003 |
|  | amputation | 168 (0.06) | 32 (0.05) | 26 (0.04) | 57 (0.08) | 53 (0.08) | <0.001 |
|  | labor pain | 82 (0.03) | 8 (0.01) | 9 (0.01) | 40 (0.06) | 25 (0.04) | < 0.0001 |
|  | others | 49142 (18.19) | 12193 (18.15) | 12107 (18.10) | 11998 (17.83) | 12844 (18.67) | < 0.0001 |
| Mental status | alert | 251269 (93.01) | 63316 (94.25) | 62553 (93.53) | 62284 (92.55) | 63116 (91.75) | 0.012 |
|  | verbal | 10190 (3.77) | 2220 (3.30) | 2332 (3.49) | 2578 (3.83) | 3060 (4.45) | < 0.0001 |
|  | pain | 7186 (2.66) | 1388 (2.07) | 1693 (2.53) | 1996 (2.97) | 2109 (3.07) | < 0.0001 |
|  | unresponsive | 1490 (0.55) | 251 (0.37) | 299 (0.45) | 437 (0.65) | 503 (0.73) | < 0.0001 |
| Vital sign | systolic blood pressure | 131.98±24.54 | 130.61 (22.61) | 130.74 (23.76) | 133.45 (25.40) | 133.04 (26.01) | < 0.0001 |
|  | diastolic blood pressure | 82.36±16.14 | 82.14 (15.16) | 81.99 (15.82) | 82.74 (16.53) | 82.55 (16.93) | < 0.0001 |
|  | pulse rate | 85.66±17.76 | 84.44 (16.14) | 85.10 (17.13) | 86.28 (18.44) | 86.75 (19.02) | < 0.0001 |
|  | respiratory rate | 17.85±4.27 | 17.68 (4.29) | 17.88 (4.26) | 17.91 (4.22) | 17.91 (4.29) | < 0.0001 |
|  | body temperature | 37.80±8.31 | 39.30 (12.67) | 38.41 (10.32) | 36.77 (2.36) | 36.74 (0.87) | < 0.0001 |
|  | oxygen saturation | 97.02±5.33 | 97.13 (5.49) | 97.17 (5.12) | 96.97 (5.35) | 96.84 (5.33) | < 0.0001 |
| Blood sugar | hypoglycemia | 727 (0.27) | 201 (0.30) | 148 (0.22) | 182 (0.27) | 196 (0.28) | 0.024 |
|  | hyperglycemia | 785 (0.29) | 207 (0.31) | 172 (0.26) | 193 (0.29) | 213 (0.31) | 0.167 |
| Emergency care | laryngeal mask airway | 164 (0.06) | 20 (0.03) | 30 (0.04) | 44 (0.07) | 70 (0.10) | < 0.0001 |
|  | oxygen administration | 36665 (13.57) | 6545 (9.74) | 7883 (11.79) | 10038 (14.92) | 12199 (17.73) | < 0.0001 |
|  | intravenous fluid infusion | 5742 (2.13) | 1157 (1.72) | 1216 (1.82) | 1558 (2.32) | 1811 (2.63) | < 0.0001 |
| Day of arrival | Monday | 39733 (14.71) | 9807 (14.60) | 9867 (14.75) | 9877 (14.68) | 10182 (14.80) | 0.035 |
|  | Tuesday | 37434 (13.86) | 9225 (13.73) | 9337 (13.96) | 9375 (13.93) | 9497 (13.81) | 0.258 |
|  | Wednesday | 37415 (13.85) | 9293 (13.83) | 9285 (13.88) | 9508 (14.13) | 9329 (13.56) | 0.320 |
|  | Thursday | 37390 (13.84) | 9282 (13.82) | 9452 (14.13) | 9253 (13.75) | 9403 (13.67) | 0.405 |
|  | Friday | 38540 (14.27) | 9507 (14.15) | 9462 (14.15) | 9725 (14.45) | 9846 (14.31) | 0.016 |
|  | Saturday | 40202 (14.88) | 10076 (15.00) | 9891 (14.79) | 9905 (14.72) | 10330 (15.02) | 0.006 |
|  | Sunday | 39425 (14.59) | 9989 (14.87) | 9583 (14.33) | 9652 (14.34) | 10201 (14.83) | < 0.0001 |
| Hour of arrival | 0 | 10830 (4.01) | 2734 (4.07) | 2642 (3.95) | 2672 (3.97) | 2782 (4.04) | 0.225 |
|  | 1 | 9676 (3.58) | 2379 (3.54) | 2325 (3.48) | 2438 (3.62) | 2534 (3.68) | 0.019 |
|  | 2 | 8112 (3.00) | 1917 (2.85) | 1963 (2.94) | 2069 (3.07) | 2163 (3.14) | <0.001 |
|  | 3 | 7513 (2.78) | 1708 (2.54) | 1775 (2.65) | 2009 (2.99) | 2021 (2.94) | < 0.0001 |
|  | 4 | 6728 (2.49) | 1542 (2.30) | 1547 (2.31) | 1736 (2.58) | 1903 (2.77) | < 0.0001 |
|  | 5 | 6759 (2.50) | 1493 (2.22) | 1603 (2.40) | 1756 (2.61) | 1907 (2.77) | < 0.0001 |
|  | 6 | 7586 (2.81) | 1731 (2.58) | 1780 (2.66) | 1931 (2.87) | 2144 (3.12) | < 0.0001 |
|  | 7 | 9476 (3.51) | 2286 (3.40) | 2294 (3.43) | 2389 (3.55) | 2507 (3.64) | 0.004 |
|  | 8 | 11824 (4.38) | 2855 (4.25) | 2980 (4.46) | 2933 (4.36) | 3056 (4.44) | 0.066 |
|  | 9 | 14521 (5.38) | 3580 (5.33) | 3614 (5.40) | 3670 (5.45) | 3657 (5.32) | 0.705 |
|  | 10 | 14606 (5.41) | 3527 (5.25) | 3612 (5.40) | 3695 (5.49) | 3772 (5.48) | 0.027 |
|  | 11 | 13141 (4.86) | 3182 (4.74) | 3289 (4.92) | 3328 (4.95) | 3342 (4.86) | 0.188 |
|  | 12 | 12281 (4.55) | 3094 (4.61) | 3076 (4.60) | 3063 (4.55) | 3048 (4.43) | 0.946 |
|  | 13 | 12227 (4.53) | 3155 (4.70) | 3058 (4.57) | 2939 (4.37) | 3075 (4.47) | 0.050 |
|  | 14 | 12499 (4.63) | 3215 (4.79) | 3200 (4.78) | 3072 (4.56) | 3012 (4.38) | 0.025 |
|  | 15 | 12349 (4.57) | 3110 (4.63) | 3123 (4.67) | 3029 (4.50) | 3087 (4.49) | 0.641 |
|  | 16 | 12009 (4.45) | 3097 (4.61) | 3127 (4.68) | 2930 (4.35) | 2855 (4.15) | <0.001 |
|  | 17 | 12036 (4.46) | 3177 (4.73) | 3036 (4.54) | 2940 (4.37) | 2883 (4.19) | <0.001 |
|  | 18 | 12675 (4.69) | 3365 (5.01) | 3181 (4.76) | 3056 (4.54) | 3073 (4.47) | <0.001 |
|  | 19 | 13417 (4.97) | 3401 (5.06) | 3370 (5.04) | 3388 (5.03) | 3258 (4.74) | 0.281 |
|  | 20 | 12774 (4.73) | 3117 (4.64) | 3239 (4.84) | 3180 (4.73) | 3238 (4.71) | 0.368 |
|  | 21 | 12707 (4.70) | 3286 (4.89) | 3082 (4.61) | 3101 (4.61) | 3238 (4.71) | 0.023 |
|  | 22 | 12448 (4.61) | 3195 (4.76) | 3000 (4.49) | 3071 (4.56) | 3182 (4.63) | 0.039 |
|  | 23 | 11945 (4.42) | 3033 (4.51) | 2961 (4.43) | 2900 (4.31) | 3051 (4.44) | 0.184 |
| Time from call to ED arrival | | 27.17±12.87 | 26.47 (12.97) | 26.12 (12.36) | 27.46 (12.75) | 28.60 (13.24) | < 0.0001 |
| Area | Gyeonggi | 63130 (23.37) | 13731 (20.44) | 14897 (22.28) | 20831 (30.95) | 13671 (19.87) | < 0.0001 |
|  | Seoul | 58686 (21.72) | 4966 (7.39) | 11847 (17.71) | 19789 (29.41) | 22084 (32.10) | < 0.0001 |
|  | Inchoen | 18178 (6.73) | 3720 (5.54) | 4145 (6.20) | 1667 (2.48) | 8646 (12.57) | < 0.0001 |
|  | Busan | 16229 (6.01) | 2650 (3.94) | 8440 (12.62) | 0 (0.00) | 5139 (7.47) | < 0.0001 |
|  | Gyeongbuk | 14298 (5.29) | 5116 (7.62) | 4796 (7.17) | 2999 (4.46) | 1387 (2.02) | < 0.0001 |
|  | Chungnam | 11779 (4.36) | 5846 (8.70) | 2392 (3.58) | 2230 (3.31) | 1311 (1.91) | < 0.0001 |
|  | Jeonbuk | 10697 (3.96) | 4117 (6.13) | 2786 (4.17) | 2363 (3.51) | 1431 (2.08) | < 0.0001 |
|  | Daegu | 10498 (3.89) | 1761 (2.62) | 532 (0.80) | 2483 (3.69) | 5722 (8.32) | < 0.0001 |
|  | Gangwon | 10267 (3.80) | 3689 (5.49) | 2757 (4.12) | 1344 (2.00) | 2477 (3.60) | < 0.0001 |
|  | Jeonnam | 9800 (3.63) | 4358 (6.49) | 4179 (6.25) | 1263 (1.88) | 0 (0.00) | < 0.0001 |
|  | Daejeon | 9935 (3.68) | 1117 (1.66) | 1553 (2.32) | 4222 (6.27) | 3043 (4.42) | < 0.0001 |
|  | Gyeongnam | 9644 (3.57) | 4943 (7.36) | 2455 (3.67) | 429 (0.64) | 1817 (2.64) | < 0.0001 |
|  | Chungbuk | 9300 (3.44) | 3021 (4.50) | 4280 (6.40) | 1999 (2.97) | 0 (0.00) | < 0.0001 |
|  | Gwangju | 6880 (2.55) | 3036 (4.52) | 1293 (1.93) | 1602 (2.38) | 949 (1.38) | < 0.0001 |
|  | Jeju | 6574 (2.43) | 3448 (5.13) | 0 (0.00) | 3126 (4.65) | 0 (0.00) | < 0.0001 |
|  | Ulsan | 4244 (1.57) | 1660 (2.47) | 525 (0.79) | 948 (1.41) | 1111 (1.62) | < 0.0001 |
| Hospital factor | hospital bed | 622.20±441.30 | 219.02±62.66 | 388.06±68.89 | 694.59±75.95 | 1172.77±479.99 | < 0.0001 |
|  | ED bed | 26.72±14.88 | 13.83±5.29 | 19.76±5.88 | 31.73±10.34 | 41.17±16.17 | < 0.0001 |
|  | ED occupancy | 0.45±0.36 | 0.24±0.26 | 0.30±0.29 | 0.55±0.31 | 0.72±0.35 | < 0.0001 |
| In-hospital cardiac arrest | | 1086 (0.40) | 150 (0.22) | 198 (0.30) | 305 (0.45) | 433 (0.63) | < 0.0001 |

ED, emergency department

**Supplementary Table 6. Performance of models predicting the occurrence of in-hospital cardiac arrest on subgroups according to the number of hospital beds**

| Model | AUROC | AUPRC | Cutoff | Sensitivity | Specificity | Accuracy | PPV | NPV |
| --- | --- | --- | --- | --- | --- | --- | --- | --- |
| Q1 | 0.9346  (0.9071, 0.9621) | 0.0910  (0.0543, 0.1487) | 0.002 | 0.8467  (0.7804, 0.8956) | 0.8987  (0.8963, 0.9009) | 0.8985  (0.8962, 0.9008) | 0.0184  (0.0171, 0.0196) | 0.9996  (0.9995, 0.9998) |
| Q2 | 0.9179  (0.8914, 0.9443) | 0.1170  (0.0791, 0.1697) | 0.002 | 0.8586  (0.8032, 0.9003) | 0.8634  (0.8607, 0.8660) | 0.8634  (0.8607, 0.8659) | 0.0183  (0.0172, 0.0194) | 0.9995  (0.9993, 0.9997) |
| Q3 | 0.9330  (0.9135, 0.9525) | 0.1404  (0.1058, 0.1841) | 0.002 | 0.9377  (0.9048, 0.9598) | 0.7889  (0.7858, 0.7919) | 0.7895  (0.7864, 0.7926) | 0.0198  (0.0192, 0.0204) | 0.9996  (0.9995, 0.9998) |
| Q4 | 0.9090  (0.8903, 0.9277) | 0.1532  (0.1222, 0.1903) | 0.002 | 0.9122  (0.8818, 0.9354) | 0.7166  (0.7132, 0.7200) | 0.7178  (0.7145, 0.7212) | 0.0200  (0.0194, 0.0206) | 0.9992  (0.9990, 0.9995) |

AUROC, area under the receiver operating characteristic curve; AUPRC, area under the precision-recall curve; PPV, positive predictive value; NPV, negative predictive value

**Supplementary Fig 1. Calibration plot of extreme gradient boosting model to predict the occurrence of in-hospital cardiac arrest**


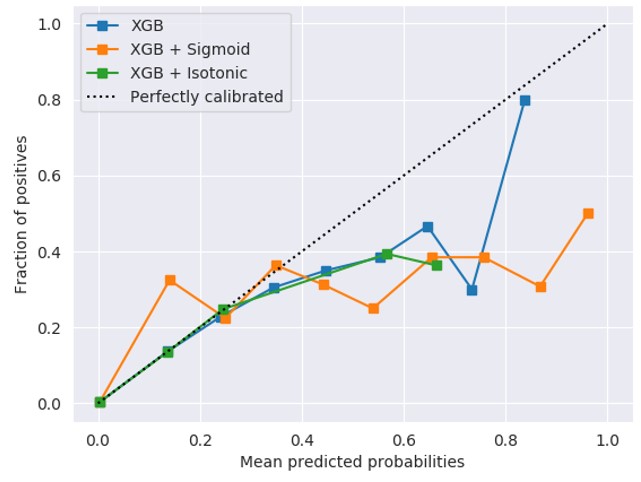


XGB, extreme gradient boosting

**Supplementary Fig 2. Performance of extreme gradient boosting model to predict the occurrence of in-hospital cardiac arrest in subgroups according to the number of hospital beds**

**
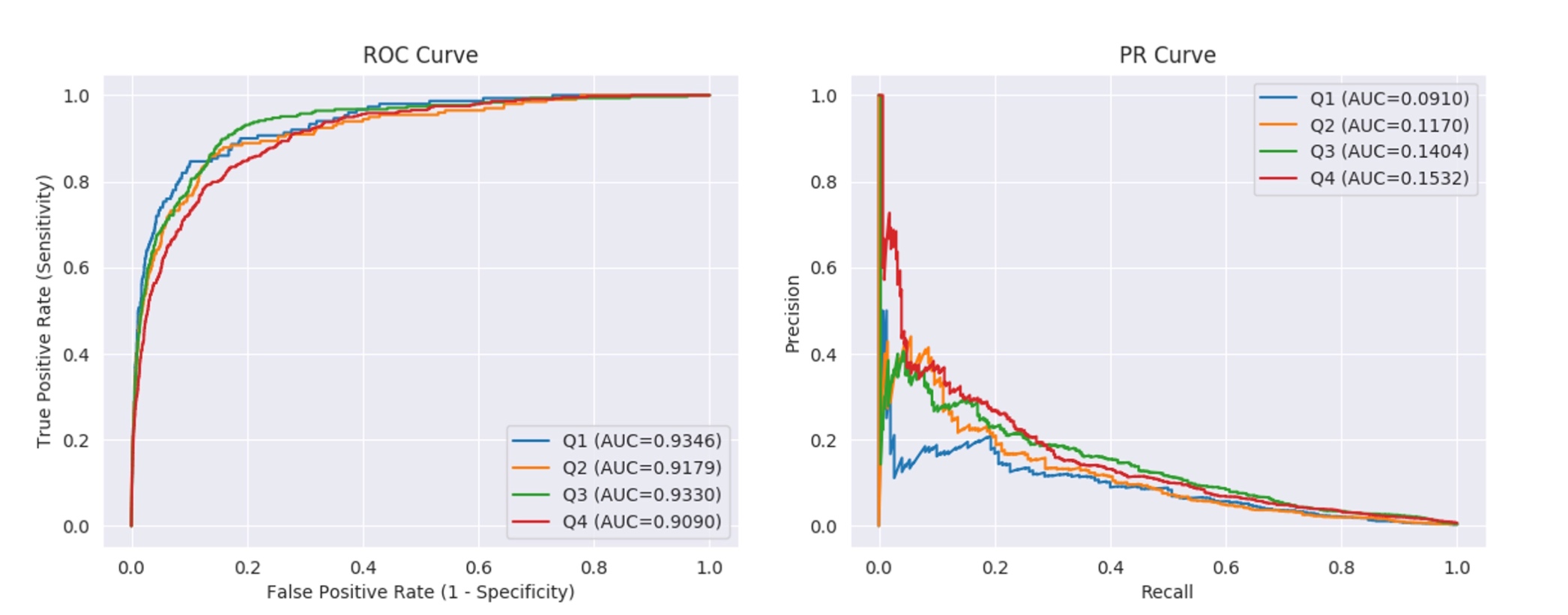
**

ROC, receiver operating characteristic; PR, precision-recall; AUC, area under the curve
